# Diagnostic value of a single β-hCG test in predicting reproductive outcomes in women undergoing cleavage embryo transfer: a retrospective analysis from a single center

**DOI:** 10.1186/s12978-022-01455-1

**Published:** 2022-06-22

**Authors:** Yuchao Zhang, Zhen Li, Bingnan Ren, Wenbin Wu, Yanli Liu, Xingling Wang, Yichun Guan, Liting Jia

**Affiliations:** 1 Department of Reproductive Medicine, The Third Affiliated Hospital of Zhengzhou University, No. 7 Kangfuqian Street, Erqi, Zhengzhou, 45005 Henan China; 2 Neonatal Screening Center, The Third Affiliated Hospital of Zhengzhou University, Zhengzhou, Henan China

**Keywords:** β-hCG, Cleavage embryo transfer, Reproductive outcomes, Cutoff values, 14 days

## Abstract

**Purpose:**

The present study investigated the role of β-hCG in predicting reproductive outcomes and established optimal β-hCG cutoff values in women undergoing cleavage embryo transfer.

**Methods:**

The patients were transferred with fresh or frozen-thawed embryos and had serum β-hCG levels tested on the 14th day post-embryo transfer. Serum β-hCG levels were compared between different groups. Different cutoff values of β-hCG were established and used to divide the patients into different groups. Reproductive outcomes between groups based on β-hCG levels were compared.

**Results:**

Significant discrepancies in general characteristics were observed in the subgroups. The cutoff values of β-hCG for predicting the presence/absence of pregnancy, biochemical pregnancy/clinical pregnancy, presence/absence of adverse pregnancy outcomes, and singleton/twin live birth in the cleavage groups were 89.6, 241.1, 585.9, and 981.1 mIU/L, respectively. Biochemical pregnancy rates and adverse pregnancy outcome rates significantly decreased from the low β-hCG group to the higher β-hCG group in sequence. Significantly higher full-term live birth rates were observed in the highest β-hCG group (P < 0.001).

**Conclusion:**

Serum β-hCG levels were strongly associated with reproductive outcomes. However, the interpretation of β-hCG levels must consider the number and quality of embryos and transfer protocols. When β-hCG was tested on a fixed day post-ET, different cutoff values were required for the prediction of early clinical outcomes. The association between β-hCG and obstetric outcomes must be investigated.

**Supplementary Information:**

The online version contains supplementary material available at 10.1186/s12978-022-01455-1.

## Plain Language Summary

To investigate the association between β-hCG and reproductive and obstetrical outcomes in women with cleavage ET and to establish different β-hCG cutoff values for the prediction of reproductive outcomes, this study retrospectively included 6909 infertile women who were divided into different groups based on the number and quality of transferred embryos, age, and transfer protocols. The cutoff values of β-hCG for predicting the presence/absence of pregnancy, biochemical pregnancy/clinical pregnancy, presence/absence of adverse pregnancy outcomes, singleton/twin live birth in the cleavage groups were 89.6, 241.1, 585.9, and 981.1 mIU/L, respectively. Biochemical pregnancy rates and adverse pregnancy outcome rates decreased significantly in the higher β-hCG groups. In conclusion, the interpretation of β-hCG levels must consider the number and quality of embryos and transfer protocols. When β-hCG was tested on a fixed day post-ET, different cutoff values were required for the prediction of early clinical outcomes.

**Supplementary Information:**

The online version contains supplementary material available at 10.1186/s12978-022-01455-1.

## Introduction

Serum beta human chorionic gonadotropin (β-hCG) has been extensively investigated, and it is a strong biomarker in predicting early pregnancy outcomes in women who are pregnant naturally or via assisted reproductive technology (ART) [1]. Higher β-hCG levels are associated with improved clinical outcomes and live birth [2–4]. Early diagnosis of adverse pregnancy outcomes (APOs), such as miscarriage and ectopic pregnancy (EP), allows effective action to prevent further damage to women. However, various factors influence β-hCG levels, and tremendous discrepancies in the included women, time, and methodologies of β-hCG measurement or protocols of embryo transfer (ET) exist between previous studies [1, 3–12]. Each laboratory should establish its own cutoff values to help physicians provide tailored treatments.

Regardless of the higher clinical pregnancy rate resulting from blastocyst transfer, the choice of cleavage embryos for transfer remains one of the main strategies in the ART procedure, especially for women with a low number of retrieved oocytes or a low opportunity for development to produce variable blastocysts. The present study investigated the association between β-hCG and reproductive and obstetrical outcomes in women with cleavage ET and established different β-hCG cutoff values for the prediction of reproductive outcomes.

## Methods

### Patients

This study was a retrospective study that initially included infertile women undergoing ART from October 2017 to September 2020 at the Department of Reproductive Medicine, the Third Affiliated Hospital of Zhengzhou University. The patients were transferred with fresh or frozen-thawed embryos. According to the department’s policy, all patients were asked to return to the department on the 14th day post-ET for β-hCG measurement. Our department followed all treated women until the ultimate clinical outcomes, such as miscarriage, EP, and live birth (LB), occurred. Patients with missing or untimely β-hCG data, more than two transferred embryos, and heterotopic pregnancies were excluded. We also excluded patients with blastocyst transfer. Because the correlation between β-hCG levels and clinical outcomes was of primary interest, whether the pregnancies achieved from embryo-derived donor egg cycles were less studied. This study was performed in accordance with the Code of Ethics in the Declaration of Helsinki, which was approved by the ethics committee of the Third Affiliated Hospital of Zhengzhou University.

### Embryo transfer

Fresh embryos or frozen-thawed embryos were transferred as previously described [13–15]. For fresh cleavage embryo transfer, two cleavage embryos were generally chosen, and a single embryo was chosen according to the patients’ condition and embryo quality. For frozen-thawed embryo transfer, the number was chosen based on the physician’s experience and patients’ condition. The most qualified embryos in any cycle were chosen for transfer. Assisted hatching (AH) was routinely performed in the frozen-thawed ET cycles and selectively performed (advanced age, previous transfer failure, or thick zona pellucid) in fresh ET cycles.

### Assessment of cleavage embryo quality

The quality of embryos was assessed on the third day post-insemination, which was clearly stated in previous studies [16]. Patients in the two embryo groups were divided into 3 subgroups based on the number of good-quality embryos (subgroup 1, good/good; subgroup 2, good/average, good/poor, average/average, average/poor; and subgroup 3, poor/poor).

### Laboratory tests

Venous blood samples were collected on the 14th morning post-ET. Samples were rested for 30 min and centrifuged at 3000 rpm for 10 min. The serum on the upper layer was used for analysis. The measurements were performed using electrochemical luminescence (ECLIA) on a Cobas 8000 (Roche Diagnostics, Germany). Daily internal quality control and yearly external quality control were performed on request. The intra-assay coefficient of variation (CV) of serum β-hCG was 2.3%, and the inter-assay CV value was 3.7%. To ensure that the data were as homogeneous as possible, we included only the data obtained from our laboratory.

### Definition of clinical outcomes

Positive β-hCG was defined as a value ≥ 5 mIU/L [4, 10], which indicated the presence of implantation. Clinical pregnancy (CP) was defined as the presence of a gestational sac and fetal heart activity following positive serum β-hCG [10]. Biochemical pregnancy (BP) was defined as a temporary rise in serum hCG without gestational sacs inside or outside the uterus [10]. BP was the pregnancy that did not end up with CP. APO included miscarriage, which was defined as a pregnancy that did not result in delivery [17], and EP was defined as CP that did not occur inside the uterine cavity [10]. LB was defined as the delivery of live babies after a gestational age of 28 weeks [13], and ongoing pregnancy (OP) was defined as a clinical pregnancy that did not result in the abovementioned outcomes [9]. Preterm birth was defined as a gestational age less than 37 weeks, and post-term birth was defined as a gestational age greater than 42 weeks [17].

### Statistical analysis

Statistical analyses were performed using Statistical Product Service Solutions version 22.0 (SPSS 22.0, IBM, Armonk, NY, USA). Normally distributed continuous variables are expressed as means (SD), and non-normally distributed continuous variables are presented as medians and inter-quartile ranges. Statistical comparisons were performed using Student’s t test or the Mann–Whitney U test where appropriate. Categorical variables are expressed as numbers or percentages, and the chi-squared test or Fisher’s exact test was used for comparisons of categorical variables where appropriate. The transfer of two embryos in women with clinical pregnancy made it impossible to evaluate the true contribution of each embryo in the very early stages of development. Therefore, further comparisons between fresh ET and frozen-thawed ET were performed only when a single embryo was transferred. A receiver operating characteristic (ROC) curve was used to calculate the optimum β-hCG cutoff values to distinguish different reproductive outcomes. The area under the curves (AUC) and their 95% confidence intervals were calculated for each ROC curve. Cutoff values were determined when the Youden index (sensitivity + specificity − 1) was the largest. The closer the percentage of ROC was to 1.0, the better β-hCG levels predicted the reproductive outcomes. The positive prediction value (PPV) and negative prediction value (NPV) were calculated for each cutoff value.

## Results

### General characteristics of included patients

**Fig. 1:**
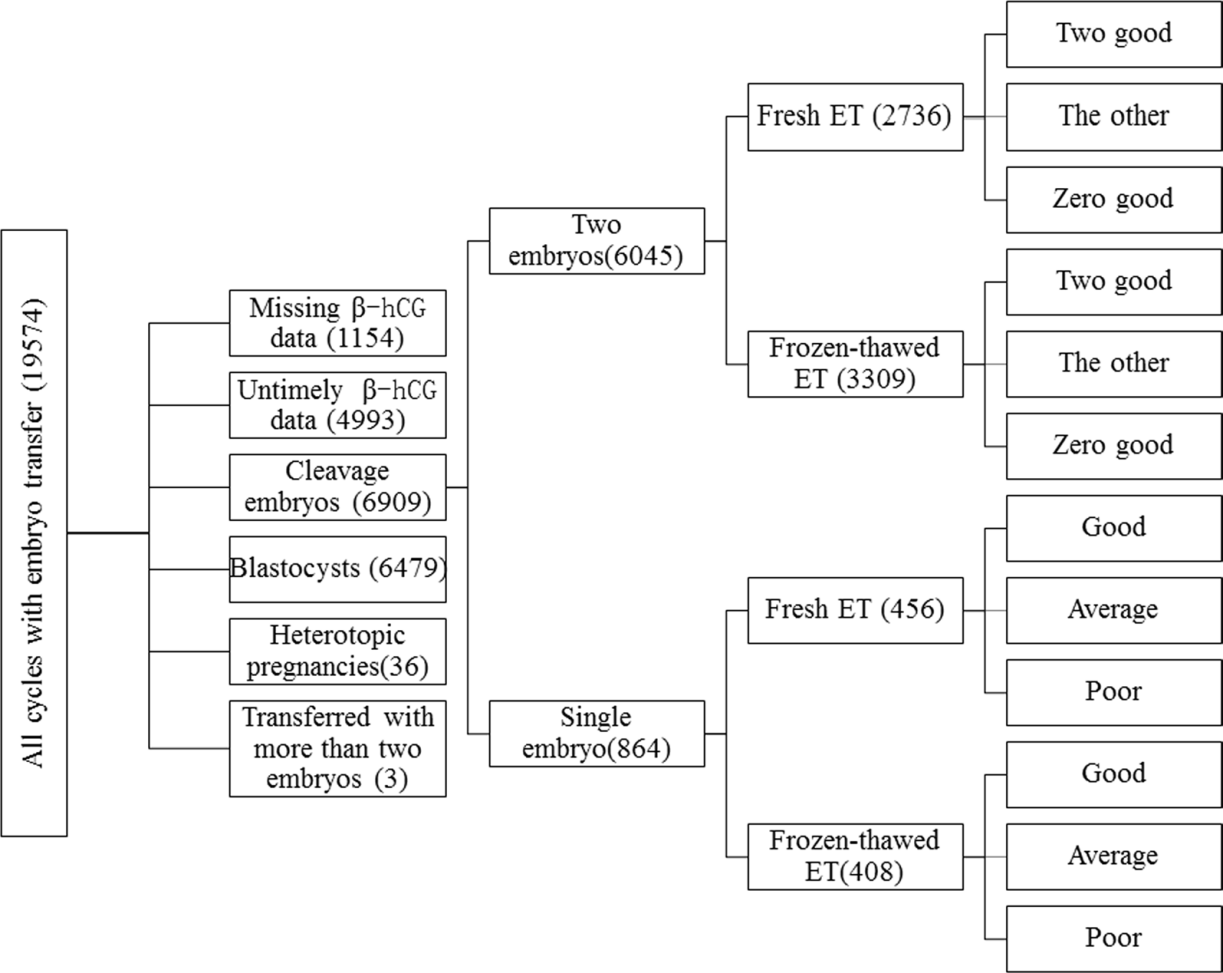
Flowchart of the included patients. Untimely β-hCG data refer to the results tested on other days instead of the 14th day post-ET. The other data refer to different combinations of the two transferred embryos, specifically, one embryo with good quality/one embryo with average quality, one embryo with good quality/one embryo with poor quality, two embryos with average quality, and one embryo with average quality/one embryo with poor quality

Figure 1 shows the flowchart of the included cycles, and Table 1 summarizes the baseline characteristics of women undergoing cleavage ET. There were 6045 cycles with two cleavage embryos and 864 cycles with a single embryo. General characteristics in age, endometrial thickness, BMI, number and quality of embryos, type and cause of infertility, and transfer protocol were compared between the single-embryo group and the two-embryo group and between the fresh and frozen-thawed ET groups when a single embryo was transferred. Significant differences were observed (Table 1).

**Table 1:** General characteristics of included patients transferred with cleavage embryos

Characteristic	Single cleavage embryo (864)				Two cleavage embryos (6045)	P
	Fresh-ET (456)	Frozen-thawed ET (408)	P	Total (864)		
Age (years) mean (SD)	32.0 (4.6)	36.3 (6.4)	< 0.001	34.0 (6.0)	32.9 (5.6)	< 0.001
Duration of infertility (years) median (IQR)	3.0 (3.5)	3.0 (4.2)	0.320	3.0 (3.3)	3.0 (3.0)	0.01
BMI (kg/m^2^) mean (SD)	24.0 (3.3)	24.4 (3.2)	0.064	24.22 (3.27)	24.0 (3.3)	0.056
Endometrial thickness (mm) (IQR)	11.0 (2.9)	9.0 (2.0)	< 0.001	10.3 (3.0)	10.0 (3.0)	0.464
Transfer protocol n (%)						
IVF/ICSI	456 (100%)	–	–	456 (52.8%)	2736 (45.3%)	< 0.001
FET	–	408 (100%)		408 (47.2%)	3309 (54.7%)	
Type of infertility n (%)						
Primary	186 (40.8%)	124 (30.4%)	0.001	310 (35.9%)	2529 (41.8%)	0.001
Secondary	270 (59.2%)	284 (69.6%)		554 64.1%)	3516 (58.2%)	
Causes of infertility n (%)						
Male factors	110 24.0%)	61 (15.0%)	< 0.001	171 (19.8%)	1212 (20.0%)	0.100
Pelvic and fallopian tube factors	153 (33.6%)	108 (26.5%)		261 (30.2%)	2004 (33.2%)	
Ovulation failure	43 (9.4%)	69 (16.9%)		112 (13.0%)	680 (11.2%)	
Other female factors	32 (7.0%)	58 (14.2%)		90 (10.4%)	495 (8.2%)	
Both side factors	91 (20.0%)	91 (22.3%)		182 (21.0%)	1268 (21.0%)	
Unexplained	27 (6.0%)	21 (5.1%)		48 (5.6%)	386 (6.4%)	
Number of top quality embryo n (%)						
0	115 (25.2%)	136 (33.3%)	0.009	251 (31.9%)	850 (15.0%)	< 0.001
1	341 (74.8%)	272 (66.7%)		613 (78.1%)	1738 (31.9%)	
2	–	–		–	3457 (63.1%)	

Data are expressed as the means (SD) or number (percentage). “–” refers to absence of data

### β-hCG levels based on the number of transferred embryos

**Fig. 2:**
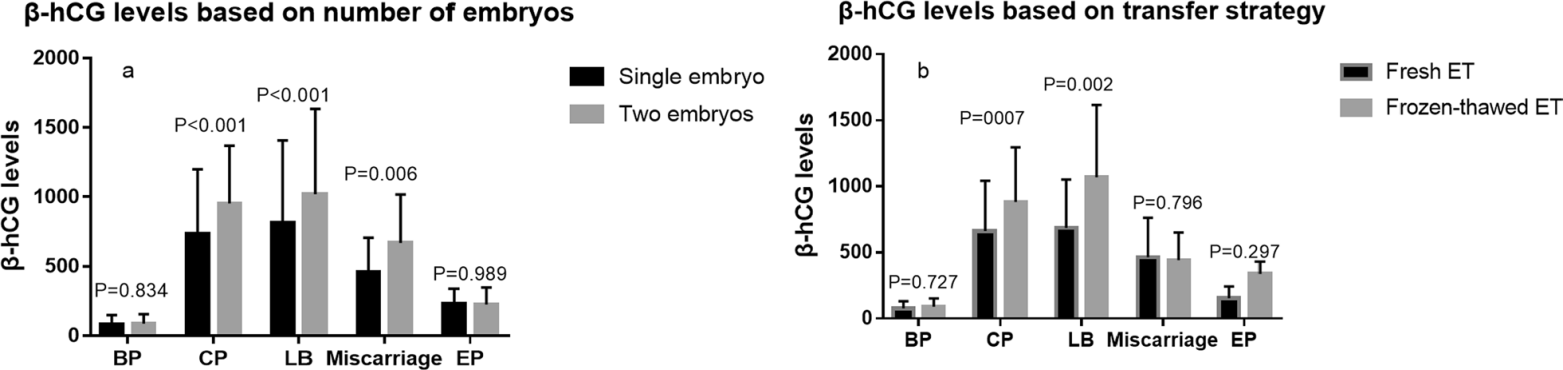
Clinical outcomes based on the number and transfer strategy of embryos

The mean β-hCG levels were similar in patients with BP (82.0 ± 67.6, 86.9 ± 68.9, P = 0.834) and EP (228.0 ± 111.5, 226.6 ± 121.5, P = 0.989) between the single-embryo group and the two-embryo group (Fig. 2a). When a single cleavage embryo was transferred in frozen-thawed ET cycles, the mean β-hCG levels were significantly higher when CP (662.4 ± 379.8, 883.2 ± 412.3, P = 0.007) and LB (685.0 ± 367.0, 1069.3 ± 545.7, P = 0.002) were achieved (Fig. 2b).

### β-hCG levels based on the quality of transferred embryos

**Fig. 3:**
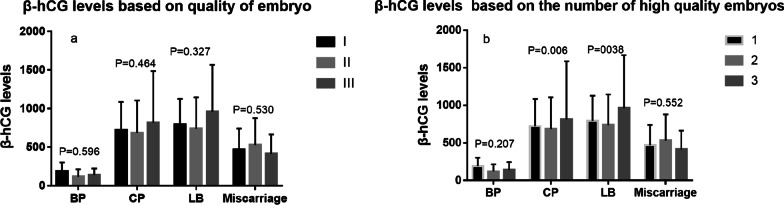
Clinical outcomes based on the quality of transferred embryos

β-hCG levels in women with a single embryo were compared according to the different quality groups. Notably, higher β-hCG levels were not significantly observed in good-quality embryos in BP, CP, LB, or miscarriage (P = 0.596, 0.464, 0.327, and 0.530, respectively) (Fig. 3a). Similar results were observed when the embryos were transferred in fresh or frozen-thawed cycles (Additional file 1: Table S2). β-hCG levels in women with two embryos were compared according to the number of good-quality embryos. Although β-hCG levels were significantly higher in women transferred with at least one average embryo, differences were only observed in women with CP and LB (P = 0.006 and 0.038) (Fig. 3b). Notably, this observation was no longer present in women  with frozen-thawed ET cycles (Additional file 1: Table S3).

### β-hCG levels based on female age

**Fig. 4:**
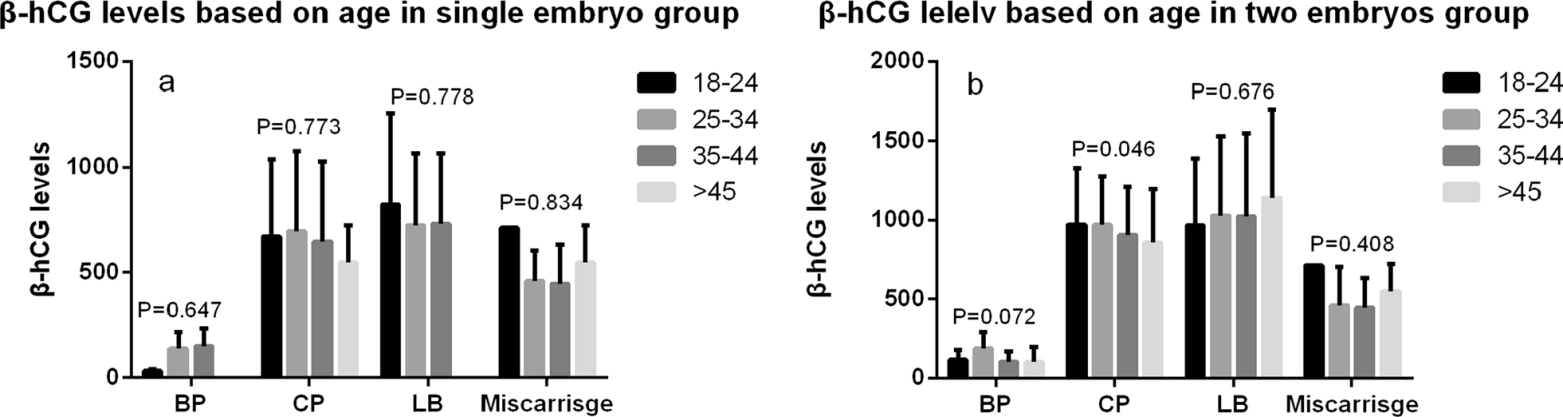
Clinical outcomes based on female age

The women were divided into 4 groups with a 10-year interval. No significant differences were observed in women with a single embryo, (Fig. 4a). We did not perform subgroup analysis due to the small sample size. β-hCG levels in women in the two-embryo group slowly but significantly decreased in women with advancing age (P = 0.046) (Fig. 4b) only when CP was achieved. Subgroup analysis showed similar results (Additional file 1: Table S4).

### Cutoff values to distinguish different reproductive outcomes

We further established crude cutoff values of β-hCG to distinguish different reproductive outcomes (Table 2) without considering the number and quality of embryos or the transfer strategy. The crude cutoff values were 89.6, 241.1, 585.9, and 981.1 mIU/L to predict the positive outcomes, with AUCs of 0.996, 0.972, 0.726, and 0.774, respectively, with sensitivity being 0.99, 0.93, 0.75, and 0.71, respectively, and with specificity being 0.97, 0.93, 0.61, and 0.70, respectively.

**Table 2:** The cutoff values to distinguish different clinical outcomes

Reproductive outcomes	Cutoff	AUC (95% CI)	P	Sensitivity	Specificity	PPV	NPV
Implantation present/absent	89.6	0.996 (0.995, 0.997)	< 0.001	0.99	0.97	97.3 (3533/3631)	98.4 (3226/3278)
BP/CP	241.1	0.972 (0.963, 0.980)	< 0.001	0.93	0.93	99.0 (3319/3352)	61.3 (423/690)
APO present/absent	585.9	0.726(0.702, 0.749)	< 0.001	0.75	0.61	90.1 (2225/2470)	34.6 (386/1115)
Singleton/twins	981.1	0.774 (0.754, 0.794)	< 0.001	0.71	0.70	48.8 (484/992)	85.6 (1196/1397)

*AUC* area under the receiver operating characteristic curve, *PPV* positive predictive value, *NPV* negative predictive value

### The application of cutoff values in the subgroups

We used the cutoff values to divide the patients into five groups. Different clinical outcomes were compared between the β-hCG-based groups, and significant differences were observed (Table 3). BP rates and APO rates decreased significantly from 87.5 to 84.2% in the lowest β-hCG group to 0.22% and 8.72% in the highest group, and CP rates and LB rates increased significantly. However, no differences were observed in gestational age, birth weight, birth length, proportion of male babies, or cesarean section rates (P = 0.249, 0.365, 0.486, 0.197, and 0.659, respectively) between the five β-hCG-based groups.

**Table 3:** The application of cutoff values in the subgroups

Groups	Cycles	BP n (%)	CP n (%)	APO n (%)	Twins n (%)	Singleton n (%)	Gestational age mean (SD)	Birth weight mean (SD)	Birth length mean (SD)	Proportion of male babies n (%)	Cesarean section rate n (%)	Post-term birth rate n (%)	Preterm birth rate n (%)
(5–89.6]	411	359 (87.4%)	52 (12.7%)	44 (84.6%)	0^a,b,c^ (0%)	5^a,b,c^ (0.3%)	39.1 (2.1)	3530.0 (739.6)	50.2 (1.5)	2 (40.0%)	4 (80.0%)	1 (20.0%)	1 (20.0%)
(89.6–241.1]	279	64 (22.9%)	215 (77.1%)	118 (54.9%)	0^c^ (0%)	76^c^ (4.5%)	38.6 (2.4)	3300.6 (716.7)	50.2 (3.2)	49 (54.5%)	57 (75.0%)	6 (7.9%)	13 (17.1%)
(241.1–585.9]	871	23 (2.6%)	848 (97.4%)	224 (26.4%)	33^c^ (6.5%)	476^c^ (27.9%)	39.0 (1.6)	3360.68 (571.2)	50.3 (1.9)	258 (54.2%)	337 (70.8%)	16 (3.4%)	42 (8.8%)
(585.9–981.1]	1114	8 (0.7%)	1106 (99.3%)	127 (11.5%)	168^b^ (20.8%)	639^b^ (37.5%)	39.0 (1.7)	3383.9 (528.0)	50.4 (2.1)	326 (51.0%)	439 (78.6%)	8 (1.3%)	41 (6.4%)
> 981.1	1368	3 (0.2%)	1365 (99.8%)	119 (8.7%)	484^a^ (48.8%)	508^a^ (29.8%)	38.9 (1.6)	3326.0 (557.2)	50.1 (2.0)	278 (54.7%)	365 (71.9%)	10 (2.0%)	47 (9.3%)
P		< 0.001	< 0.001	< 0.001	< 0.001		0.249	0.365	0.486	0.197	0.659	< 0.001	

Data are expressed as the means (SD) or number (percentage)

^a, b, c^Different letters indicate statistical significance between the two groups

## Discussion

Although the role of serum β-hCG in predicting early pregnancy outcomes was extensively investigated, large differences exist between previous studies. Each laboratory should identify its own optimistic β-hCG levels. Patients who underwent cleavage ET and had β-hCG detected on the 14th day post-ET were included in this retrospective study. In addition to the comparisons of β-hCG levels between different groups, we further calculated different cutoff values of β-hCG to predict early pregnancy outcomes.

The combination of β-hCG and progesterone has been used to predict the prognosis of pregnancy outcomes in women who conceived naturally or via ART [12, 18]. According to clinical experience, pregnant women undergo a large fluctuation in serum progesterone levels. Therefore, we did not measure the progesterone levels on the β-hCG testing day. We found that serum β-hCG alone predicted early pregnancy outcomes in women who underwent cleavage embryo transfer.

Serum β-hCG levels are measured on a fixed day post-oocyte retrieval in some reproductive departments, and one β-hCG level might fit all. However, Zhang et al. reported [19] that β-hCG levels in pregnancies resulting from day 5 transfers were lower than day 3 transfers, which suggested that delayed embryo transfer impaired embryo development or implantation potential [19]. Theocharis et al. [4] found the same trend but no statistical significance, and claimed that β-hCG levels were highly predictive of pregnancy outcome after blastocyst transfer compared to cleavage embryo transfer. Although Wang et al. [8] claimed a strong predictive role of β-hCG levels on a fixed day post-ET, they did not report the average β-hCG levels in the subgroups [8]. Our results seemed relatively homogeneous without considering blastocysts. β-hCG levels were reported based on the number and quality of cleavage embryos. These differences were consistent when a single fresh or frozen-thawed cleavage embryo was transferred.

Two cleavage embryos are often chosen for transfer in clinical practice. Because the vanishing twin phenomenon affects maternal serum β-hCG levels, we compared the β-hCG levels between single- and two-embryo transfers in the subgroups and found similar β-hCG levels in women who ended up with BP or EP. Twofold higher β-hCG levels were not found in women transferred with two embryos compared to women with a single embryo (except for women with one or two transferred blastocysts and ended up with twin live birth). Our results support the theory that the vanishing embryo implants but fails to develop and only slightly contributed to the β-hCG levels in the early stage.

Inconsistent β-hCG levels were reported after fresh or frozen-thawed ET in previous studies [3, 5, 6, 11]. The testing day post-ET, embryo biopsy, method of fertilization, method of freezing and thawing, media of embryo culture, and etiology of infertility may explain the discrepancies. To maximally reduce the confounding factors, we further analyzed the β-hCG levels in patients who were transferred with ranked embryos. According to our policy, good-quality embryos were primarily chosen for transfer in fresh cycles, and we observed higher rates of top-quality embryos and thicker endometrium in fresh ET, which contributed to higher CP rates. However, when a pregnancy was achieved, higher β-hCG levels were observed in the frozen-thawed ET cycles. One hypothesis based on our clinical practice was that the embryos in frozen-thawed cycles were subjected to AH, which may contribute to earlier implantation and made the blastocyst produce more β-hCG. However, we cannot explain the association between a significantly thicker endometrium and low β-hCG levels in fresh ET cycles. One study supported an association between young age and lower initial hCG values after ART treatment regardless of the mode of treatment [20]. However, we did not find the same result. The most likely explanations were the transferred embryos and β-hCG measurement in the two studies.

Different cutoff values of β-hCG were established to predict the clinical outcomes in previous studies [3, 5, 6, 8, 9]. Xu et al. [10] further established a mathematical model for predicting EP, which reduced the medical resources spent on women with low EP risk and provided targeted tailor-made treatment for women with a higher risk of EP. Because of the tremendous discrepancies between the studies, each department should establish its own cutoff value. The present study established different cutoff values of β-hCG for cleavage embryos. Excellent performance of the cutoff values was shown in both groups, which suggested that clinicians could adopt convincing cutoff values to make clinical decisions. However, serious caution should also be taken because we observed live births in women whose β-hCG levels were as low as 52.66 IU/L but increased rapidly a few days later (Additional file 1: Table S1).

Few studies addressed the association between β-hCG levels and obstetric outcomes in ET cycles. Therefore, we ultimately divided the women into different groups according to the cutoff values of β-hCG and observed a decreased full-term live birth rate in women who were transferred with cleavage embryos in the low β-hCG group. These results suggested that β-hCG was associated with short-term clinical outcomes, such as BP, CP, and EP and long-term obstetric outcomes, such as gestational age. However, the exact mechanism behind the phenomenon is not defined. One hypothesis is that embryos with top quality were associated with a higher CP rate and LB rates, and embryos that were implanted secrete more hCG. However, hCG prevents trophoblast cells from attack by maternal lymphocytes and stimulate fetal testis to secrete testosterone, which promotes sexual differentiation and gonad development. The hCG ultimately promotes the aromatization of androgen into estrogen and stimulates the formation of progesterone. In general, hCG keeps pregnancy stable until live birth.

There were some limitations in our study. (1) β-hCG levels in further subgroups, such as the PGT group and usual blastocyst group, and embryos with top and average quality were not compared. (2) The number and quality of transferred embryos were not strictly restricted, which may make our data suitable for a more wider population under the condition of a large sample size. (3) The β-hCG levels following the 14th day were not included or analyzed. According to clinical practice, we only asked patients to have more β-hCG measurements when the first result was relatively low. The purpose of this study was to determine the levels at which the patients should be asked for follow-up β-hCG, which would simplify decision-making for clinicians and reduce the unnecessary burden for patients. (4) Because pregnancy after ART is a high-risk pregnancy, additional data should be available on other gestational complications (e.g., preeclampsia, GDM, IUGR, and IUFD) to correctly support the hypothesis that early β-HCG values have a long-lasting predictive value in advanced gestation.

The strengths of our study include (1) the large sample size, which may make the results more convincing, and (2) comparison of obstetric outcomes in different β-hCG groups. Significant differences existed when the women were pregnant and gave LB, which suggested a long-term association with β-hCG groups 14 days post-ET.

## Conclusions

Serum β-hCG levels were strongly associated with reproductive outcomes. However, the interpretation of β-hCG levels must consider the number and quality of embryos and transfer protocols. When β–hCG was tested on a fixed day post-ET, different cutoff values were required for the prediction of early clinical outcomes. The association between β-hCG and obstetric outcomes must be investigated.

## Supplementary Information

**Additional file 1: Table S1.** The women who gavelive birth but had the lowest β-hCG levels. **Table S2.**β-hCG levels based on the quality of single transferred embryo. **Table S3.** β-hCG levels based on thenumber of good quality embryo in women with two embryo. **Table S4.** β-hCG levels based on women age in two embryos group.

## Data Availability

The data used during the current study are available from the corresponding author upon reasonable request.

## Abbreviations

β-hCG: Beta-human chorionic gonadotropin
ART: Assisted reproductive technology
APO: Adverse pregnancy outcomes
EP: Ectopic pregnancy
ET: Embryo transfer
LB: Live birth
AH: Assisted hatching
ECLIA: Electrochemical luminescence
CV: Coefficients of variation
CP: Clinical pregnancy
BP: Biochemical pregnancy
OP: Ongoing pregnancy
ROC: Receiver operating characteristic
AUC: Area under the curve
PPV: Positive prediction value
NPV: Negative prediction value
