# Supplementary material for: Diagnostic value of a single β-hCG test in predicting reproductive outcomes in women undergoing cleavage embryo transfer: a retrospective analysis from a single center
Source: Reprod Health. 2022 Jun 22;19:145. doi: 10.1186/s12978-022-01455-1 (PMC9215108; doi:10.1186/s12978-022-01455-1)
Supplement: Supplementary file 1 — Additional file 1: Table S1. The women who gavelive birth but had the lowest β-hCG levels. Table S2.β-hCG levels based on the quality of single transferred embryo. Table S3. β-hCG levels based on thenumber of good quality embryo in women with two embryo. Table S4. β-hCG levels based on women age in two embryos group. [file 12978_2022_1455_MOESM1_ESM.docx]

Table S1 The women who gave live birth but had the lowest β-hCG levels

| Age (years) | Quality | The days post embryo transfer (mIU/L) | | | | | |
| --- | --- | --- | --- | --- | --- | --- | --- |
|  |  | 14 day | 16 day | 17 day | 18 day | 19 day | 20 day |
| 29 | 2 top | 52.66 |  |  |  | 790.4 |  |
| 26 | 2 top | 65.7 |  |  |  |  | 814 |
| 30 | 2 top | 76.6 |  | 704.6 |  |  |  |
| 41 | 1 top 1 average | 78.47 | 172.6 |  |  | 845.3 |  |
| 28 | 1 top | 85.14 | 190.6 |  | 406.6 |  |  |
| 33 | 1 top | 92.52 |  |  | 986.6 |  |  |

Excellent rise of β-hCG levels were observed in the following tests.

Table S2 β-hCG levels based on the quality of single transferred embryo

| Quality | BP | | CP | | LB | | Miscarriage | |
| --- | --- | --- | --- | --- | --- | --- | --- | --- |
|  | Fresh ET | Frozen-thawed ET | Fresh ET | Frozen-thawed ET | Fresh ET | Frozen-thawed ET | Fresh ET | Frozen-thawed ET |
| I | 226.19±79.53 | 117.03±66.03 | 685.8±231.49 | 804.65±417.2 | 724.12±304.23 | 956.18±355.96 | 476.37±297.8 | 456.99±245.06 |
| II | 117.26±69.98 | 119.66±71.51 | 650.94±323.7 | 781.18±402.32 | 700.51±421.09 | 821.1±374.74 | 518.24±313.95 | 556.38±465.27 |
| III | 41.73±25.96 | 218.33±85.16 | 559.41±267.68 | 1081.3±1757.01 | 549.86±274.07 | 1372.2±2055.34 | 498.05±251.07 | 359.35±210.42 |
| P | 0.195 | 0.582 | 0.278 | 0.439 | 0.154 | 0.302 | 0.928 | 0.488 |

Table S3 β-hCG levels based on the number of good quality embryo in women with two embryo

| Number | BP | | CP | | LB | | Miscarriage | |
| --- | --- | --- | --- | --- | --- | --- | --- | --- |
|  | Fresh ET | Frozen-thawed ET | Fresh ET | Frozen-thawed ET | Fresh ET | Frozen-thawed ET | Fresh ET | Frozen-thawed ET |
| Two good | 125.67±75.08 | 134.99±67.58 | 942.07±338.6 | 1067.83±549.03 | 985.57±413.02 | 1178.07±482.28 | 633.93±311.19 | 815.72±316.43 |
| The other | 107.95±59.93 | 216.12±138.66 | 862.69±313.05 | 1042.31±478.96 | 915.15±311.59 | 1148.23±466.21 | 622.89±369.34 | 702.76±305.54 |
| Zero good | 115.88±44.49 | 55.24±42.65 | 766.3±269.6 | 945.05±420.91 | 787.36±436.61 | 1088.65±433.6 | 726.68±421.35 | 553.41±262.58 |
| P | 0.85 | 0.128 | 0.000 | 0.219 | 0.001 | 0.585 | 0.634 | 0.162 |

| Table S4 β-hCG levels based on women age in two embryos group   \| Age \| BP \| \| CP \| \| LB \| \| Miscarriage \| \| \| --- \| --- \| --- \| --- \| --- \| --- \| --- \| --- \| --- \| \| Fresh ET \| Frozen-thawed ET \| Fresh ET \| Frozen-thawed ET \| Fresh ET \| Frozen-thawed ET \| Fresh ET \| Frozen-thawed ET \| \| 18-24 \| 126.73±78.66 \| 99.89±76.98 \| 954.6±388.42 \| 999.68±307.83 \| 930.24±544.54 \| 1020.47±488.52 \| 1019.65±428.16 \| 571.5 \| \| 25-34 \| 116.19±63.12 \| 286.93±114.31 \| 889.61±317.49 \| 1077.68±484.84 \| 931.64±498.77 \| 1161.02±574.5 \| 618.22±346.72 \| 745.99±432.7 \| \| 35-44 \| 106.27±58.17 \| 101.2±52.37 \| 810.35±222.2 \| 976.37±326.12 \| 888.76±538.45 \| 1130.07±602.71 \| 628.62±352.66 \| 682.73±371.08 \| \| >45 \| 18.83± \| 142.27±58.29 \| 790.33±162.21 \| 863.75±366.34 \| 790.33±162.21 \| 1247.83±619.73 \| - \| 668.79±381.58 \| \| P \| 0.847 \| 0.017 \| 0.032 \| 0.024 \| 0.651 \| 0.544 \| 0.086 \| 0.818 \| |
| --- | --- | --- | --- | --- | --- | --- | --- | --- | --- | --- | --- | --- | --- | --- | --- | --- | --- | --- | --- | --- | --- | --- | --- | --- | --- | --- | --- | --- | --- | --- | --- | --- | --- | --- | --- | --- | --- | --- | --- | --- | --- | --- | --- | --- | --- | --- | --- | --- | --- | --- | --- | --- | --- | --- | --- | --- | --- | --- | --- | --- | --- | --- |
|  |
